# Impact of tobacco-pack pictorial warnings on youth and young adults: A systematic review of experimental studies

**DOI:** 10.18332/tid/108614

**Published:** 2019-05-15

**Authors:** Diane B. Francis, Nia Mason, Jennifer Cornacchione Ross, Seth M. Noar

**Affiliations:** 1 Department of Communication, University of Kentucky, Lexington, United States; 2 Manship School of Mass Communication, Louisiana State University, Baton Rouge, United States; 3 Department of Social Sciences and Health Policy, Wake Forest School of Medicine, Winston Salem, United States; 4 School of Media & Journalism, University of North Carolina at Chapel Hill, Chapel Hill, United States; 5 Lineberger Comprehensive Cancer Center, University of North Carolina at Chapel Hill, Chapel Hill, United States

**Keywords:** systematic review, youth, tobacco control, pictorial warning labels

## Abstract

**INTRODUCTION:**

We conducted a systematic review of the experimental literature on the impact of tobacco-pack pictorial warning labels (PWLs) on youth and young adults.

**METHODS:**

We systematically searched computerized databases and the reference lists of relevant articles. We included studies that used an experimental protocol to assess PWLs. Studies had to report findings for youth or young adult samples (aged <30 years). Thirty-one studies met the inclusion criteria, with a total sample size of 27506. Two coders independently coded all study characteristics and outcomes.

**RESULTS:**

Twenty-eight studies experimentally evaluated PWLs for cigarette packs while three studies evaluated PWLs for smokeless tobacco packs. Generally, PWLs led to higher attention, stronger cognitive and affective reactions, more negative pack attitudes and smoking attitudes, and increased intentions not to use tobacco products compared to text warnings. PWLs were perceived to be more effective than text warnings for both cigarette packs and smokeless tobacco packs.

**CONCLUSIONS:**

The systematic review showed that PWLs on tobacco products are effective across a wide range of tobacco-related outcomes among young people. Gaps in the literature include a lack of research on tobacco initiation and cessation and a dearth of literature on non-cigarette tobacco products.

## INTRODUCTION

Tobacco use is the leading cause of preventable death globally^1^, accounting for more than 7 million deaths each year^2^. The majority of these deaths are caused by cigarette smoking^2^, which is the most common form of tobacco use in most countries. Globally, the number of young people aged 13–15 years who smoke cigarettes is estimated at 25 million^1^. The median smoking prevalence among those aged 13–15 years across 61 countries is estimated at 10.7%^3^. Moreover, the smoking prevalence among this age group exceeded 20% in several countries, including Argentina, Italy, and Jordan^3^.

Most cigarette smoking begins during adolescence and continues into adulthood^3^. In the United States, for example, almost 90% of cigarette smokers first try smoking by the age of 18 years and 98% by the age of 26 years^4^. Nearly one in three adults who have ever smoked cigarettes began smoking daily between 18–26 years of age^4^. Furthermore, two-thirds of young people who try smoking become daily smokers^5^. Consequently, for young people, just trying smoking is a significant risk factor for long-term use. The increasing age of smoking initiation and the high likelihood of conversion of ever smokers to daily smokers make young people a critically important population group for tobacco prevention and control^4,5^.

Use of non-cigarette tobacco products (NCTPs, e.g. smokeless tobacco) has also increased sharply in the past decade, especially among young people. Globally, 13 million youth aged 13–15 years use NCTPs, including smokeless tobacco^1^. Use of other NCTPs is exceptionally high in some low- to middle-income countries. Nepal, for example, has a high prevalence of smokeless tobacco use among young people. In 2011, 19.7% of boys and 12.9% of girls aged 13–15 years used smokeless tobacco^1^. Nicotine addiction is associated with a higher risk of lifetime tobacco use^4^. As such, there remains a continued need for evidence on effective approaches to communicating the health risks of tobacco use among young people and preventing tobacco use among this vulnerable population group^4^.

Pictorial warning labels (PWLs) on tobacco product packaging constitute an effective tobacco prevention and control policy. PWLs are effective in communicating the health risks of tobacco use^6-9^. As of 2018, 78 countries, representing 47% of the world’s population, met best practices for PWLs, which includes printing the warnings in the local language and covering an average of at least half of the front and back of cigarette packs^10^. Between 2014 and 2016, 34 countries including India and Bangladesh, with a total of 2 billion people adopted large graphic PWLs^1^. India and Bangladesh both require warnings on cigarette and smokeless tobacco packs.

Systematic reviews and meta-analyses have shown that relative to text-only warnings, like those still in use in the USA, PWLs are significantly more effective across a range of tobacco-related outcomes^6-9^. The evidence is clear and consistent, that there is a direct association between cigarette PWLs and increased cessation, reduced smoking initiation, and prevalence^8,11-15^. PWLs also are associated with increased attention to warnings, negative affective reactions, knowledge about health risks, quitting behaviors, and reduced susceptibility to tobacco use^7-9^.

However, while many studies have been published on PWLs, the impact of such warnings on young people remains under-studied^16^. For instance, the Noar et al.^9^ meta-analysis of experimental PWL studies found that 11% focused only on adolescent samples. In addition, adolescents comprised 12– 14% of the samples in two reviews of observational PWL studies^7,8^. While many studies in those reviews included people in the 18–30 years age range, studies rarely separated the effects among these younger age groups. No previous systematic review has solely examined youth populations, and very little is known about PWLs for NCTPs such as smokeless tobacco^16^. Similar to prior research with adult samples, we reviewed experimental studies because these studies compare pictorial to text or control warnings. Thus, this systematic review aimed to investigate the extent to which PWLs impact on extant outcomes among young people. The study adds to understanding the effects of PWLs on adolescents and young adults.

## METHODS

### Search strategy and study selection

We used a systematic search strategy to locate all peer-reviewed studies on PWLs with youth and young adult samples. We searched five computerized databases in February 2017 and updated the search in January 2018^1^. The databases were: Medline, PsycINFO, Communication and Mass Media Complete, Web of Science, and Business Source Complete. We chose these databases because they were used in previous reviews to locate relevant PWL studies^9,17^. As the systematic review initially focused on PWLs for all tobacco products, we used the following search string: (cigarette* OR tobacco OR smok* OR smokeless OR waterpipe OR hookah OR shisha OR cigar* OR snuff OR pipe OR e-cig OR vape OR little cigar OR snus OR e-cigarette OR electronic cigarette OR electronic nicotine delivery system OR ENDS OR chewing tobacco OR chew OR loose leaf OR dip OR dissolvable tobacco OR novel tobacco) AND (warning* OR label* OR packag* OR pictorial OR graphic) AND (youth* OR adolescent* OR young OR teen* OR student* OR kid* OR children OR young adult*).

We examined references of seven published PWL reviews and meta-analyses^6-9,16,18,19^. We searched the first 100 results of our search terms in Google Scholar. Once we identified the final set of articles for the review, we examined all references in those articles to look for relevant studies to potentially be included.

To be included in the review, a study had to: be a within-subjects or between-subjects experimental design; have a pictorial condition and a text or control condition; report effects separately for youth/young adults (<30 years); and be available in English^9^. Although one study^20^ did not report ages for their study samples, we included them in the review because their study was of school-age samples. We excluded studies on PWLs tested solely in media campaigns or on advertisements. We also excluded quasi-experimental designs that showed participants the warnings but did not have a control condition (e.g. Adebiyi^21^, Hawari^22^, and Goodall^23^). We also excluded observational studies that asked people to report on warnings they saw on their own before being exposed during the study (e.g. Baskerville^24^).

**Figure 1:**
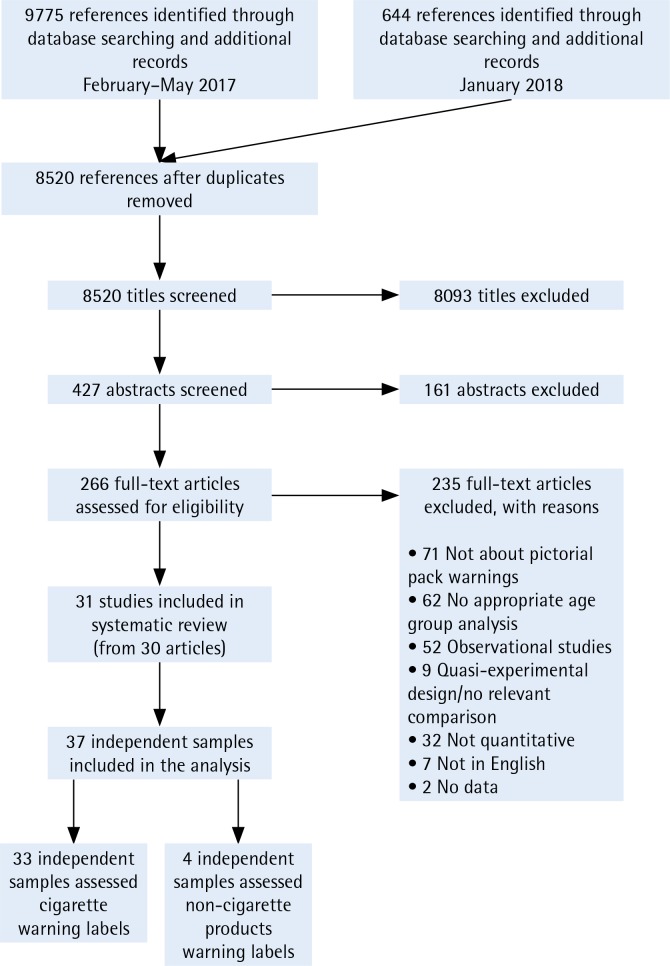
PRISMA flow diagram showing the study screening process

Two coders independently applied the inclusion criteria described above throughout the screening process. The coders first screened all titles and abstracts and then reviewed full-text articles for relevance. They tracked reasons for exclusion of full-text articles not included in the review. The combined searches in 2017 and 2018 yielded 8520 references after removing duplicates. After screening titles and abstracts, 266 full-text articles were reviewed. The screening and review process resulted in 31 studies and 37 independent samples for the systematic review (Supplementary file). Figure 1 shows the PRISMA flow diagram that demonstrates the study screening process.

### Data extraction and article coding

Two coders independently coded sampling, study design and warning characteristics for each study in the review. We categorized study outcomes according to the message impact framework (MIF), which describes factors contributing to PWL effectiveness^9,17^. The framework draws on communication and psychological theory and prior research on tobacco prevention and control to posit the communication process. The framework includes six major categories: attention and recall; warning characteristics; knowledge, attitudes, and beliefs; intentions; behavior; and social interactions. Perceived effectiveness was also examined. Each category includes a set of constructs relevant to the mechanisms by which PWLs exert their influence (see Noar et al.^9^ for detailed definitions of each construct). During the coding process, discrepancies were resolved first between the coders. If discrepancies remained unresolved, the coders consulted with other team members for clarification. Mean per cent agreement across all coding categories was 94% and Cohen’s κ had a mean value of 0.92.

## RESULTS

### Study characteristics

Table 1 shows a summary of study characteristics. Thirty-one studies were included in the analysis for this review^20,25-53^. Studies were published between 2000 and 2017, with a median publication year of 2015. The studies included samples from 13 countries: 20 studies were conducted in the USA^20,25,27-29,31-34,36,40-46,49,53^, four in Canada^30,35,48,49^, two in Germany^50,52^, and one each in 10 other countries. Sample sizes ranged from 19 to 9183; the total sample size across all studies was 27506.

**Table 1:** Characteristics of studies in the systematic review (k=31)

*Variable*	*k*	*%*
**Age groups**		
Adolescents only	14	45
Young adults only	13	42
Adolescents & young adults	4	13
**Cigarette smoking status**		
Smokers	9	29
Non-smokers	1	3
Smokers and non-smokers	21	68
**Smokeless tobacco use**		
Smokeless tobacco	6	19
NR	26	84
**Country**		
USA	20	65
Canada	4	13
Germany	2	6
Other countries each studied once (Bangladesh, China, France, Greece, India, Lebanon, Mexico, The Netherlands, Spain, Switzerland)	10	32
**Sampling**		
Probability	3	10
Convenience	28	90

Many numbers add up to over k=31 or over 100% because some studies included multiple options for each characteristic. Some studies were conducted in multiple countries. NR: not reported.

Thirty-one studies were included in the analysis for this review^20,25-53^. Studies were published between 2000 and 2017, with a median publication year of 2015. The studies included samples from 13 countries: 20 studies were conducted in the USA^20,25,27-29,31-34,36,40-46,49,53^, four in Canada^30,35,48,49^, two in Germany^50,52^, and one each in 10 other countries. Sample sizes ranged from 19 to 9183; the total sample size across all studies was 27506.

Fourteen studies (47%) sampled youth only (≤18 years)^20,27,29,32,33,35,37,38,42,45,46,48,51^ and 13 (42%) examined young adults only (18–30 years)^30,31,34,36,39,40,44,50,53^. Males represented 47% of the samples across all studies. Among the 14 studies (45%) reporting race and ethnicity—all from the USA—Whites represented 59% of the samples, Black/African Americans were 22%, Hispanics were 15%, Asians were 12%, American Indians were 2%, and other or mixed races were 13%^20,25,28,29,31-34,36,40,41,43,45,46^.

Regarding methodological approach, 17 studies (55%) used a between-subjects design where participants were randomized to the PWL or text/ control conditions; the other studies used a within-subjects design. The majority of studies used convenience sampling (90%), with the remaining 10% using probability sampling. Participants were recruited in a variety of ways, including: through the internet (46%); community settings (29%); elementary, middle and high schools (22%); and colleges and universities (17%). Twelve studies (39%) used theory to guide their research. Theories used included psychological reactance^40,53^, cognitive dissonance^52^, and the common-sense model^53^, among others.

Twenty-eight studies (90%) assessed PWLs for cigarette packages^20,26-29,31-53^, while three studies (10%) assessed PWLs for smokeless tobacco^25,30,54^. Only three studies (10%) placed warnings on actual tobacco packs, as most of the remaining studies presented the warnings on a computer screen (i.e. digital). Almost all studies tested warnings already in use in the study country or other countries. Table 2 shows a summary of PWL characteristics.

**Table 2:** Characteristics of PWLs in studies in the systematic review

*Variable*	*k*	*%*
**Product assessed**		
Cigarette	28	90
Smokeless tobacco products	3	10
**Number of different warnings viewed**		
1	8	26
2–64	23	74
**Number of times viewed each warning**		
1 time	24	77
2–5 times	4	13
NR	3	10
**Exposure channel**		
Digital	26	84
Cigarette pack	3	10
Printed or paper	2	6
NR	1	3
**Warning size**		
30% of the pack	1	3
50% of the pack	7	23
NR	22	71
**Type of pack**		
Branded	13	42
Generic	8	26
Plain	2	6
NR	11	35
**Label order**		
Random	16	52
Non-random	6	19
NR	1	3
n/a (1 label or all shown at once)	8	26
**Nature of graphic warnings**		
Image only	3	10
Image with text	28	90

During the initial search in 2017, we ran the search terms across several databases, including Medline and PubMed. We decided to use Medline only for the final search because the process did not reveal any differences between the two databases. NR: not reported.

### Impact of PWLs for cigarette packs

**Figure 2:**
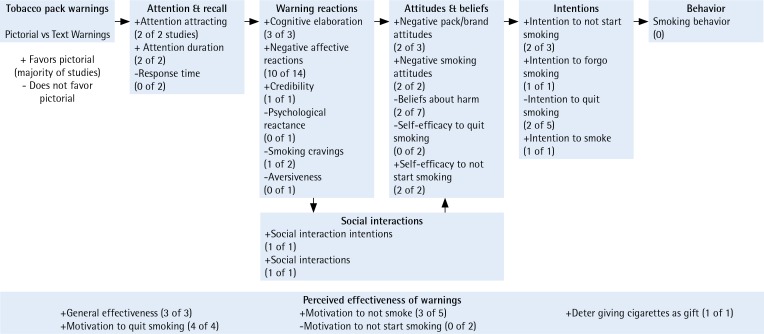
Effects of pictorial warnings on tobacco packs (summary of findings)

We next summarize key findings, organizing them according to the message impact framework categories (Figure 2)^9^. With a few exceptions^26,43^, most studies reported findings for overall youth and/or young adult samples. Where appropriate or when overall samples were not reported on, we report details for samples stratified by various sociodemographic characteristics.

#### Attention

Six studies assessed attention to the warnings on cigarette packs^26,29,37,39,45,52^. Attention-attracting^26,45^ and attention duration^29,39^ were both assessed in two studies each. Attention attraction is the extent to which the warning attracted or grabbed participants’ attention. Attention duration is the amount of time participants spent viewing the warning label. PWLs elicited significantly higher attention across both studies. Moreover, the results held when analyses were stratified by gender and smoking status^26^. Response time is the amount of time it took participants to complete questions or click forward after viewing the warning label. Response time was not significantly different in the two studies that assessed this variable^37,52^.

#### Warning reactions

Seventeen studies assessed warning reactions^20,26,27,29,31-34,39,40,43-45,50,51,53^. Overall, PWLs produced a range of warning reactions, including cognitive elaboration and negative affective reactions. Three studies assessed cognitive elaboration (i.e. thinking about the health risks of smoking). PWLs were significantly more effective in eliciting cognitive elaboration among young people compared to text or control warnings in those studies^27,40,51^. Fourteen studies assessed negative affective reactions^26,27,29,31,33,34,39,40,43-45,50,53^. For the majority of studies, exposure to PWLs resulted in more negative affective reactions than exposure to text or control warnings^26,27,31,40,43-45,50,53^. In one study, female youth had significantly higher scores than male youth for negative affective reactions^43^; Black and Hispanic youth also had significantly higher scores among young adults on negative affective reactions^43^. Credibility was significantly higher for PWLs than the text control in one study^20^. A study examining psychological reactance found no statistically significant difference^45^; aversiveness was also not statistically significant^34^. The findings were mixed for smoking cravings; one study found significant differences after exposure to PWLs^27^ while another found no differences^32^.

#### Attitudes and beliefs

Thirteen studies evaluated attitudes and beliefs^26,27,29,33,35,41,43,45,46,48-50,52^. PWLs were significantly more effective at eliciting negative pack/brand attitudes^27,49^ and smoking attitudes^35,41^ among youth and young adults relative to text or control warnings. However, one study found that negative pack/brand attitudes favored text warnings^48^. PWLs did not change beliefs about smoking harms across the seven studies that assessed this outcome^26,29,33,43,45,46,50,52^. Two studies found females had higher beliefs about harms than males^26,43^ and one found young African Americans and Hispanics had higher beliefs about harms than Whites^43^. PWLs influenced self-efficacy to quit or not start smoking^26,50^.

#### Behavioral intentions

Seven studies assessed behavioral intentions or the likelihood of quitting, not starting or forgoing smoking^26,28,29,33-35,48^. Three studies assessed intentions not to start smoking^26,35,48^, and PWLs were found more effective at eliciting these intentions in two of three studies^26,35^. In one study examining intentions to forgo cigarettes, PWLs were significantly more effective at eliciting intentions compared to text warnings^34^. Five studies assessed intentions to quit smoking, with mixed results^26,28,29,33,34^. Two studies found significant differences favoring PWLs^28,34^, two studies found no significant differences^26,29^ between PWLs and text, and one study found significant differences favoring the text condition over PWL^33^. PWL reduced intentions to smoke in one study^28^.

#### Social interactions

One study assessed social interaction intentions^26^ and another assessed actual social interactions^45^. Both studies found PWLs to be significantly more effective compared to text or control warnings at persuading young people to talk to others about the warning.

#### Perceived effectiveness

Perceived effectiveness is concerned with participants’ perceptions of the effectiveness of warning messages. Sixteen studies assessed perceived effectiveness of the warnings^20,26,31,34,36,38,40,43-47,50,51,53^. PWLs were rated higher in general effectiveness in three studies^26,34,38^. Four studies assessed the impact of warnings on motivation to quit smoking; all found PWLs to be significantly more effective for young people compared to text or control warnings^36,44,47,50^. Of the five studies that assessed motivation not to smoke^31,45,46,53^, PWLs were significantly more effective in three studies^31,53^. In two studies, PWLs were rated significantly less effective in motivation to not start smoking compared to text warnings^44,51^. One study found significant effects that PWLs are perceived to be effective in deterring giving cigarettes as a gift^47^.

### Impact of PWLs for smokeless tobacco products

Three studies evaluated the effect of PWLs for smokeless tobacco products, resulting in four independent samples^25,30,42^. Perceived effectiveness was the main outcome assessed in those studies. Young people rated PWLs on smokeless tobacco as significantly more generally effective^25,42^ and less appealing^25,30^ than text warnings or controls.

## DISCUSSION

This study contributes to the growing evidence of the effectiveness of PWLs across different population groups. After synthesizing the findings from 31 studies with almost 30000 young people, we found that PWLs elicited greater attention, stronger cognitive reactions (thinking about harms) and negative affective reactions (e.g. fear, disgust), more negative pack attitudes and smoking attitudes, and increased intentions to not use tobacco products. Findings are similar to previous systematic reviews with primarily adult populations^7-9^ and suggest that PWLs are vital in communicating health risks of tobacco use and potentially moving young people away from initiating tobacco use or towards quitting tobacco products. In summary, the studies examined in this review showed encouraging evidence of effects of PWLs on young people that discourage them from using tobacco products.

Although the findings on most outcomes generally support the greater effectiveness of PWLs, the findings on behavioral intentions were mixed, and the findings on beliefs about smoking harms were not significant. In our review, PWLs elicited more cognitive elaboration and negative emotional reactions. This is consistent with what appear to be the active mechanisms of PWLs’ impact among adult smokers^55^. On the other hand, we found mixed results for intentions and no effect for beliefs about smoking harms, findings also consistent with prior review studies^9^. The results for intentions may be due to inadequate measures^17^. Researchers have pointed out the importance of including timeframes when asking about intentions, arguing that intention to change in the next month is different from intention to change in the next six months. The lone study reporting a timeframe for intentions found a significant effect of PWLs for intentions to quit smoking in the next week. Given that this review examined studies of adolescents and young adults, asking about a more immediate timeframe may be more relevant to them than asking about long-term intentions to quit. Or, it may be that brief exposure to warnings is not always enough to change intentions, but repeated exposures over time will lead to intention change^56^. Regarding beliefs about smoking harms, evidence is beginning to amass that risk beliefs — especially cognitively-oriented beliefs such as perceived likelihood of harm — play little to no role in the impact of PWLs^9,55^. Instead, such warnings seem to have an impact by eliciting in-themoment affective arousal and cognitive elaboration, as we have found in this review. Still, more careful studies on the effects of PWLs are required before we can make stronger conclusions regarding extant risk beliefs and intentions in warning effectiveness among adolescents and young adults.

In this review, countries with the highest prevalence of overall tobacco use among young people were rarely represented in the research. Nepal, for example, has some of the highest prevalence of smokeless tobacco use among adolescent boys and girls^3^. Argentina has high rates of cigarette smoking^3^. However, none of the studies in the review was conducted with samples from these countries. Two of the three studies on non-cigarette tobacco products were in countries with high rates of non-cigarette tobacco use (India and Bangladesh). However, more studies are needed to determine the impact of PWLs on non-cigarette tobacco products as a tobacco control strategy for young people.

Tobacco use is often a social behavior. Young people whose parents are smokers are at high risk for initiation. PWLs on parents’ packs are seen by children, which also may broaden their impact in various ways^45,57^. However, we need more work in this area as most studies have been focused at the level of the individual. Only one study in this review evaluated the impact of youth exposure to warnings on their parents’ packs^45^. Similarly, studies are increasingly examining social interactions that take place around warnings^58-60^, recognizing that warnings can spark conversations with a variety of people that may play a role in their impact. Future research could examine a variety of dyadic and social processes that may influence PWL impact.

Despite evidence to the contrary, young people maintain an optimistic bias towards smoking^61,62^. Young tobacco users often do not connect tobacco use to long-term health problems^61^. In one study, 60% of adolescents smokers believed they could smoke for a few more years and then quit with no adverse health effects, compared to 48% of adult smokers^62^. Potentially due to its long-term, far-off consequences, young tobacco users do not see the link between tobacco use and many chronic diseases. That said, countries have a limited set of warnings, despite having adolescent, young adult, and adult populations. To maximize the impact of warnings on youth and young adults, we should ensure that we implement content that resonates with younger population groups, in addition to adult smokers. While the main target of PWLs may be adult smokers (and cessation behavior), young people are a critical secondary audience for tobacco warnings.

In this review, we observed an increase in the number of studies focusing on youth and young adult populations throughout the study period: 24 of the 31 studies (77%) were published in 2010 or later. However, the majority of studies in our review were about PWLs for cigarettes. So far, studies on the effectiveness of PWLs for NCTPs have not kept pace with their rapid rise in use. Use of NCTPs has surged in the USA and other western countries in the past five years, especially among young people^1,5^. Increased use of NCTPs makes identifying evidence-based strategies to communicate the health risks of non-cigarette tobacco products an urgent priority^16,63^. Thus, more studies are needed that focus on PWLs for NCTPs (e.g. little cigars, waterpipe tobacco) and not only smokeless tobacco products. The limited evidence that we have suggests that PWLs seem to operate similarly for cigarettes and non-cigarette tobacco products. Nevertheless, we need more studies to identify specific warnings to implement because some NCTPs have different health effects, and specific research is needed to guide both warning content and format.

### Limitations and future directions

The study has several limitations. First, we were unable to draw conclusions on the effects of PWLs to stem tobacco use initiation among young people. A previous review pointed out the limited research on smoking initiation^9^, and this is still the case. Young people in this review did perceive that warnings would motivate them not to start tobacco use, but rigorous studies examining the impact on initiation have not been undertaken. Perceived effectiveness that warnings would stop one from initiating cigarettes is not the same as actual effectiveness^64^. Longitudinal studies could attempt to quantify the number of young people prevented from using cigarettes and non-cigarette tobacco products due to the warnings. Second, the review did not evaluate the impact of real-world observational studies; those studies could also examine to what extent PWLs aid in reducing the prevalence of tobacco initiation and use. To date, there are few observational studies on adolescents.

Consequently, as more countries implement PWLs, it is imperative to conduct systematic evaluations to assess their effectiveness among young people. The choice to focus on published literature and studies available in English could be one reason for the limited number of studies from developing countries. More research from these high-prevalence areas would contribute to answering questions related to how PWLs work and for whom they work. Finally, a meta-analysis to quantitatively assess the impact of PWLs on extant outcomes among young people is warranted.

## CONCLUSIONS

Preventing tobacco use among young people is critical to ending the tobacco epidemic worldwide. Moreover, PWLs on tobacco products are a vital part of those tobacco control efforts. As such, it is essential to evaluate the evidence for PWLs among young people. Much of what we know about the effectiveness of such warnings come from studies of adult populations. Youth and young adults represent key target population groups for PWLs. This systematic review sought to expand our understanding of the impact of tobacco pack PWLs on tobacco-related outcomes for young people. We showed that PWLs on tobacco products are effective across a wide range of outcomes, including attention, negative affective reactions, and perceived effectiveness. More work is needed to bolster the impact of warnings on young people and to estimate the effect of warnings to reduce tobacco initiation among young people. Continued research will also further build the evidence base that PWLs communicate health risks of smoking and other forms of tobacco use.
